# Novel mechanisms for crotonaldehyde-induced lung edema

**DOI:** 10.18632/oncotarget.17840

**Published:** 2017-05-12

**Authors:** Yue Li, Jianjun Chang, Yong Cui, Runzhen Zhao, Yan Ding, Yapeng Hou, Zhiyu Zhou, Hong-Long Ji, Hongguang Nie

**Affiliations:** ^1^ Institute of Metabolic Disease Research and Drug Development, China Medical University, Shenyang 110122, Liaoning, China; ^2^ Department of Anesthesiology, First Affiliated Hospital of China Medical University, Shenyang 110001, Liaoning, China; ^3^ Department of Cellular and Molecular Biology, University of Texas Health Science Center at Tyler, Tyler, Texas 75708, USA; ^4^ Texas Lung Injury Institute, University of Texas Health Northeast, Tyler, Texas 75708, USA

**Keywords:** epithelial sodium channels, crotonaldehyde, lung injury, alveolar fluid clearance, reactive oxygen species

## Abstract

**Background:**

Crotonaldehyde is a highly noxious α,β-unsaturated aldehyde in cigarette smoke that causes edematous acute lung injury.

**Objective:**

To understand how crotonaldehyde impairs lung function, we examined its effects on human epithelial sodium channels (ENaC), which are major contributors to alveolar fluid clearance.

**Methods:**

We studied alveolar fluid clearance in C57 mice and ENaC activity was examined in H441 cells. Expression of α- and γ-ENaC was measured at protein and mRNA levels by western blot and real-time PCR, respectively. Intracellular ROS levels were detected by the dichlorofluorescein assay. Heterologous αβγ-ENaC activity was observed in an oocyte model.

**Results:**

Our results showed that crotonaldehyde reduced transalveolar fluid clearance in mice. Furthermore, ENaC activity in H441 cells was inhibited by crotonaldehyde dose-dependently. Expression of α- and γ-subunits of ENaC was decreased at the protein and mRNA level in H441 cells exposed to crotonaldehyde, which was probably mediated by the increase in phosphorylated extracellular signal-regulated protein kinases 1 and 2. ROS levels increased time-dependently in cells exposed to crotonaldehyde. Heterologous αβγ-ENaC activity was rapidly eliminated by crotonaldehyde.

**Conclusion:**

Our findings suggest that crotonaldehyde causes edematous acute lung injury by eliminating ENaC activity at least partly via facilitating the phosphorylation of extracellular signal-regulated protein kinases 1 and 2 signal molecules. Long-term exposure may decrease the expression of ENaC subunits and damage the cell membrane integrity, as well as increase the levels of cellular ROS products.

## INTRODUCTION

In the lung, a thin layer of fluid is critical for efficient gas exchange on the apical surface of the alveolar epithelium [1, 2], and lung edema is resolved by active salt and water transport [3]. Epithelial sodium (Na^+^) channels (ENaC) are responsible for the majority of Na^+^ transport across the apical membranes of alveolar epithelial cells [1, 4, 5], and increasing evidence has shown that ENaC, which is inhibited by the drug amiloride, appears to be essential in lung fluid clearance [6, 7]. Five ENaC subunits have been cloned to date, namely, α-, β-, γ-, δ- and ε-ENaC [8, 9], and, when expressed in heterologous systems, the amiloride sensitivity and other properties of cloned αβγ-ENaC reflect of the characteristic of highly Na^+^ selective of the channels found in native epithelial cells [8]. The α-subunit is believed to form electrically detectable Na^+^-selective channels, whereas the β- and γ-subunits serve as accessory regulatory subunits that can increase, by two orders of magnitude, the amplitude of channel activity from the α-subunit alone [10]. Moreover, neonatal mice deficient in α-ENaC die shortly after birth with fluid-filled lungs [11, 12], and polymorphisms of SCNN1A, which encodes the α-subunit, may play important roles in the susceptibility to respiratory distress syndrome, particularly in term infants [13].

Cigarette smoke has been associated with a variety of harmful effects on the lung that can result in lung dysfunction, ranging from inflammatory disorders to tumors [14–21]. Previous studies showed that addition of cigarette smoke condensate in culture medium reduced the expression of α ENaC subunit at the mRNA level in immortalized tumorous human alveolar epithelial cell lines [22]. In our recent paper, we studied the effects of formaldehyde, a well-known and common pollutant, on ENaC, and determined that formaldehyde can decrease ENaC activity and contribute to edematous acute lung injury by reducing transalveolar Na^+^ transport [23]. Experimental studies suggest that α,β-unsaturated aldehydes in cigarette smoke may lead to chronic obstructive pulmonary disease, acute lung injury, asthma, and lung cancer [17, 24–27]. Among these aldehydes, acrolein has been shown to augment interleukin-8 mRNA stability via p38 mitogen-activated protein kinase (MAPK) signaling in human pulmonary cells [28].

Crotonaldehyde (CRO), known to have adverse effects on respiratory health, is a major component of cigarette smoke and a ubiquitous environmental pollutant [29]. Nevertheless, the effects of CRO on lung fluid transport have not been examined. In this study, we test the hypothesis that CRO depresses ENaC as a mechanism of aldehyde-induced edematous acute lung injury. As models for examining ENaC activity, we used the human epithelial lung cell line H441, Xenopus oocytes expressing human αβγ-ENaC, and C57 mice. We also examined the effects of CRO on the extracellular signal-regulated protein kinases 1 and 2 (ERK1/2) in our system; when activated, these enzymes are known to inhibit ENaC activity by phosphorylating residues in the C-termini of the β and γ subunits, which enhances the docking of the ubiquitin ligase (Nedd4-2) with these subunits [23]. Our results showed that CRO reduced mouse transalveolar fluid clearance *in vivo* and inhibited ENaC activity acutely by facilitating the phosphorylation of ERK1/2. Additionally, long-term exposure to CRO resulted in decreased the expression of ENaC subunits, the accumulation of products of reactive oxygen species (ROS) in cytosol, and damage to cell membrane integrity.

## RESULTS

### CRO decreases mouse alveolar fluid clearance *in vivo*

**Figure 1:**
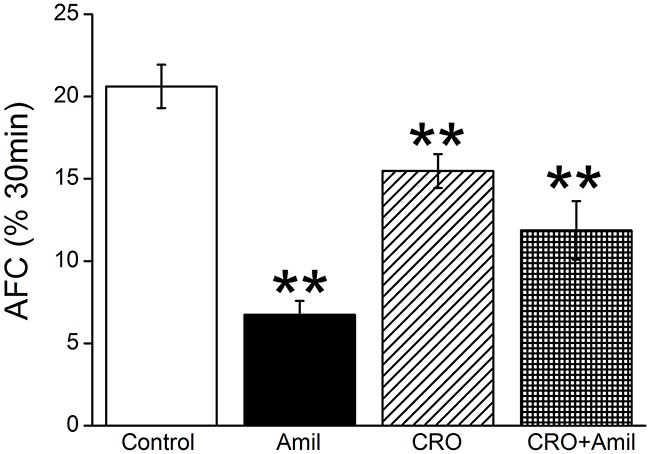
CRO downregulates mouse alveolar fluid clearance *in vivo* Anesthetized mice were intratracheally instilled with 5% bovine serum albumin dissolved in physiologic saline solution, administered alone for the control group (Control) or containing amiloride (Amil, 1mM), crotonaldehyde (CRO, 80 μM), or both (CRO+Amil) for exposed mice. The reabsorption rate was computed as the percentage of instilled volume after 30 min (% 30min). Average AFC values are presented as mean ± SE, one-way ANOVA. ^**^P < 0.01, compared with control group, n = 8.

To examine the impact of CRO on fluid resolution in mouse lungs, alveolar fluid clearance (AFC) was evaluated in anesthetized C57 mice by measuring the reabsorption of an instillate of 5% bovine serum albumin (BSA). As shown in Figure 1, the normal AFC rate was 20.6 ± 1.3% (n = 8), whereas in the presence of 1 mM amiloride, the rate was reduced to 6.7 ± 0.8% (*P* < 0.01 versus Control). Intratracheal instillation of CRO (80 μM) reduced the reabsorption of BSA to 15.5 ± 1.0%, a level similar to that found when both amiloride and CRO were administered (11.9 ± 1.8%, *P* < 0.01 versus Control), indicating that CRO was almost entirely responsible for the inhibition of the ENaC-associated amiloride-sensitive fraction of AFC. One millimolar concentration of amiloride was used in the AFC setup because a large fraction of amiloride becomes protein bound, and a significant fraction rapidly leaves the air spaces due to its low molecular weight, thus the effective alveolar concentration was probably lower. In addition, the same amiloride concentration has been used in other studies of AFC in both developing and adult animals [30, 31]. Our results showed that the AFC in the Amil + CRO group appeared greater than that in the presence of Amil alone. CRO is a strong oxidant, which could oxidize amiloride and in turn reduces the inhibition efficiency of Amil on ENaC component of AFC. These data strongly indicate that CRO impairs transalveolar fluid clearance contributed by ENaC, which conversely leads to fluid accumulation in lung.

### CRO inhibits amiloride-sensitive short-circuit currents in H441 monolayers

**Figure 2:**
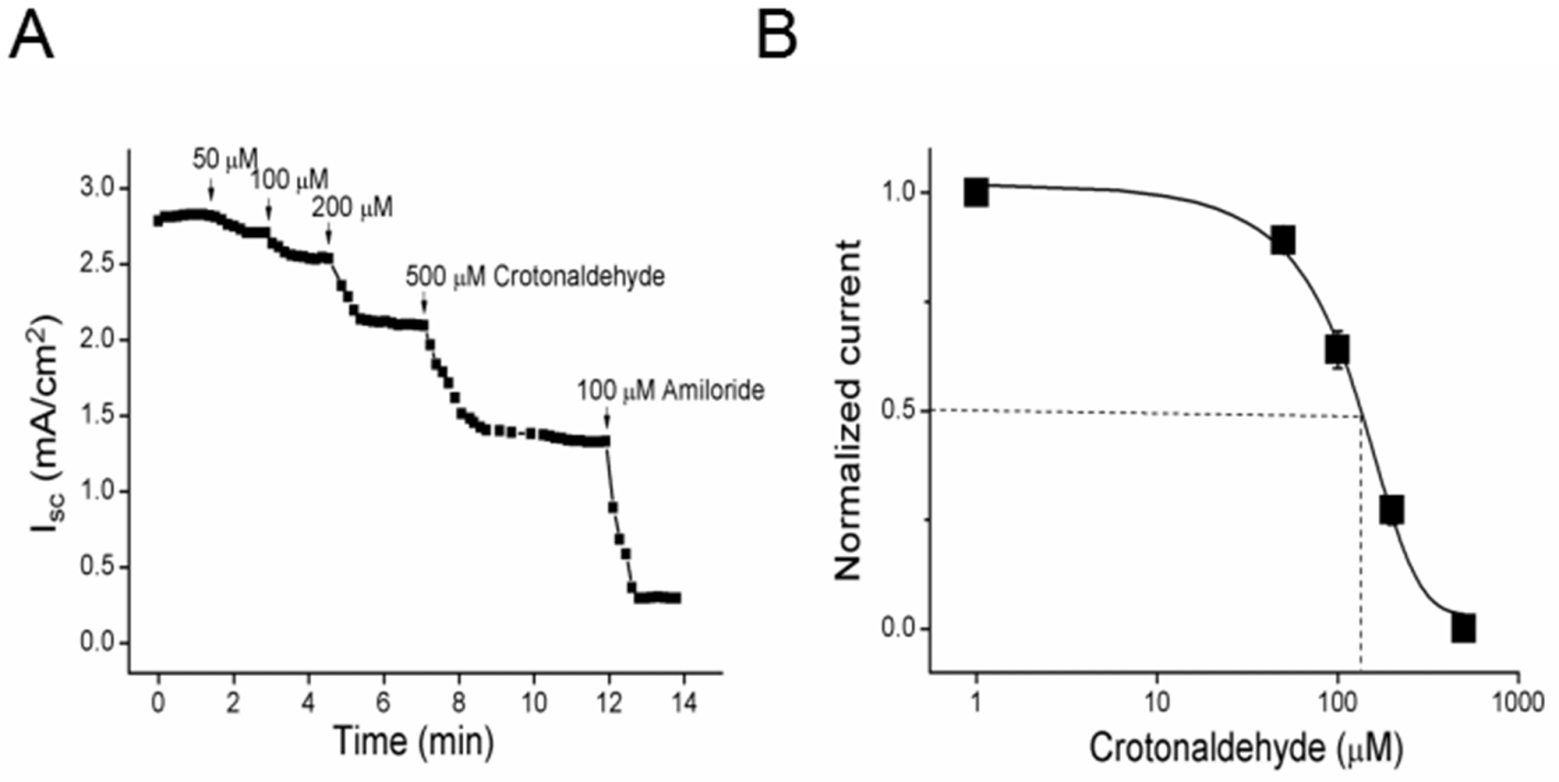
Short-circuit current level in H441 monolayers is reduced by CRO in a dose-dependent manner **(A)** Representative short-circuit current (I_sc_) traces after treatment of monolayers with 50, 100, 200, or 500 μM CRO, and amiloride was then applied. Amiloride-sensitive currents are defined as the difference between the total current and the amiloride-resistant current, with the basal amiloride-sensitive current set as 100%. **(B)** Normalized amiloride-sensitive currents were plotted as a dose-dependent curve (n = 9). The raw data were calculated by fitting with the Boltzmann equation, and the IC_50_ value was calculated to be 108.1 μM.

The amiloride-sensitive component of AFC is contributed by ENaC-mediated fluid transport, with ENaC reportedly contributing to ~60% of AFC [32]. We postulated that ENaC was a target of CRO-induced lung edema. To examine how CRO affects the electrogenic transepithelial Na^+^ transport, confluent H441 monolayers were mounted in an 8-chamber Ussing chamber system. The result indicated that CRO inhibited short-circuit currents (Isc) levels dose-dependently, and the currents could be significantly inhibited by 100 μM amiloride, a specific inhibitor of ENaC (Figure 2A). We defined amiloride-sensitive currents as the difference between the total current and the amiloride-resistant current and set the initial amiloride-sensitive current as 100%. After H441 monolayers were treated with 50, 100, 200, and 500 μM CRO, the amiloride-sensitive Isc decreased to 94.6 ± 1.3%, 83.1 ± 2.7%, 68.7 ± 3.1% and 48.4 ± 4.9%, respectively. The IC_50_ value, i.e., the half-maximal inhibitory concentration, was 108.1 ± 25.2 μM and was calculated by fitting the dose-response curve with the Boltzmann equation [33] (Figure 2B). Overall, these findings indicated that CRO treatment decreased ENaC activity.

### CRO inhibits the protein expression of α- and γ-ENaC subunits in H441 cells

**Figure 3:**
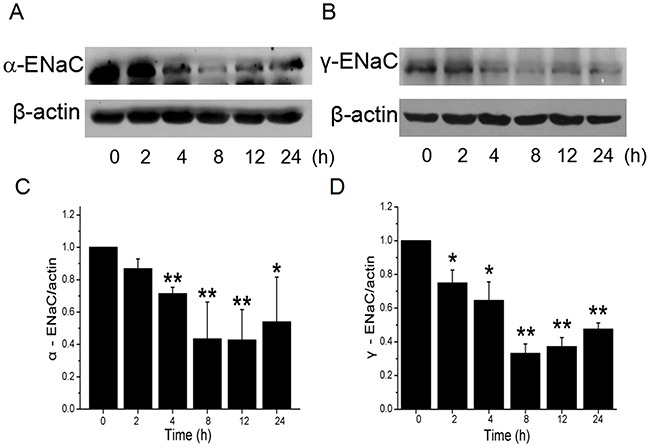
Effects of CRO on the protein expression level of ENaC α- and γ-subunits in H441 cells H441 cells were exposed to 80 μM CRO for 0 to 24 h and then proteins were extracted and analysed by western blot. **(A, B)** Western blots of α- and γ-ENaC protein demonstrating reductions in levels over time. Blots for β-actin were used as internal controls. **(C, D)** Graphical representation of data obtained from three sets of western blot assays for which bands were quantified using gray analysis (α-ENaC/β-actin and γ-ENaC/β-actin). Data are shown as the mean ± SE, ^*^P < 0.05, ^**^P < 0.01, compared with control.

H441 cells were treated with 80 μM CRO for 2, 4, 8, 12 and 24 h. CRO treatment reduced the protein levels of both subunits (Figure 3A and 3B). With β-actin expression set as an internal standard, the specific band about 97 kD for the α-ENaC protein could be seen according to the manufacturer's manual and the full-length blots/gels were presented in Supplementary Figure 1. Our analyses revealed that α-ENaC protein reached minimum levels at 8 h following CRO treatment, and then increased very slightly, but with levels remaining significantly lower than that of the time zero control (Figure 3C). The pattern observed with γ-ENaC protein (Figure 3D) was similar to that of α-ENaC (full-length blots/gels presented in Supplementary Figure 2). We did not examine β-ENaC protein expression in cell lysates because a suitable anti-β ENaC antibody was not available for the western blot assay.

### CRO depresses mRNA levels of α- and γ-ENaC subunits in H441 cells

**Figure 4:**
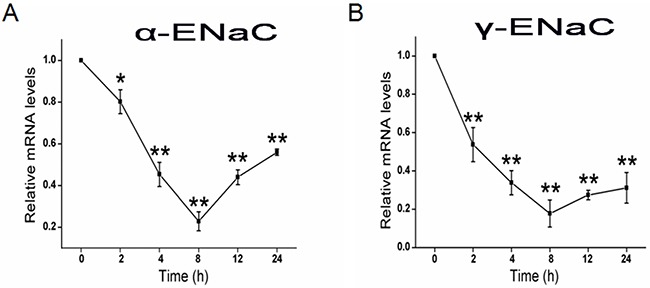
CRO reduces the transcriptional expression of ENaC α- and γ-subunits H441 cells were treated with CRO for 0 to 24 h and then RNA samples were isolated for real-time PCR assays. **(A, B)** Relative levels of mRNA were calculated as α- or γ-ENaC/GAPDH ratios. ^*^P < 0.05, ^**^P < 0.01, compared with control; average of 3 experiments for each type of subunit.

As CRO reduced ENaC protein expression levels, we predicted a possible decrease at the transcriptional level of ENaC subunits. We pretreated H441 cells with 80 μM CRO for different periods and then examined the levels of α- and γ-ENaC mRNA expression by real-time PCR. Results indicated that α-ENaC mRNA level decreased rapidly, with significant decreases already detectable by 2 h (Figure 4A); by 8 h, levels had dropped to 22.8 ± 4.5% of the levels in the control cells. As shown in Figure 4B, γ-ENaC mRNA expression was also suppressed quickly, falling to 17.8 ± 7.0% of control levels by 8 h. Taken together, these data demonstrated time-dependent inhibition of α- and γ-ENaC expression at the protein and transcriptional levels following CRO exposure of H441 cells.

### CRO increases ERK1/2 phosphorylation

**Figure 5:**
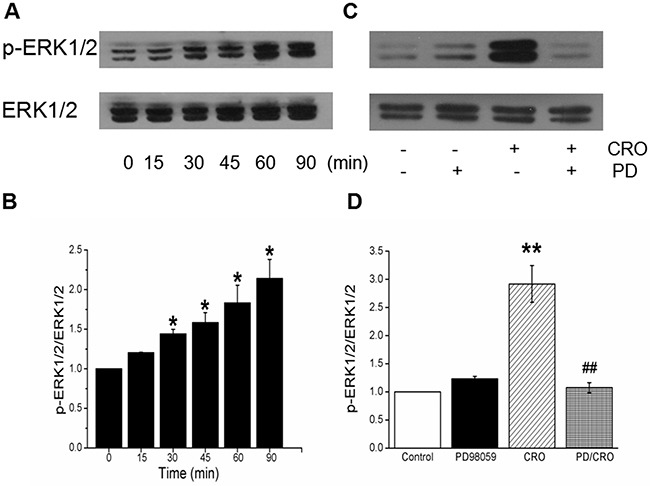
CRO elevates ERK1/2 phosphorylation level in H441 cells, and PD98059 inhibits CRO-induced ERK1/2 phosphorylation **(A)** Western blots assays for ERK1/2 phosphorylation in H441 cells exposed to 80 μM CRO for 0 to 90 min. **(B)** Graphical representation of data obtained from three sets of western blot assays for which bands were quantified using gray analysis (p-ERK1/2/ERK1/2). **(C)** Representative western blot of phosphorylated ERK1/2 in untreated H441 cells (Control), after pretreatment with 40 μM PD98059 (PD98059), after treatment with 80 μM CRO (CRO), and after pretreatment with PD98059 for 30 min prior to the addition of CRO (PD98059/CRO). Lanes shown in this figure are from the same western blot. **(D)** Ratio of the band densities for p-ERK1/2 and ERK1/2 in H441 cells. Data were obtained using three western blots and are shown as the mean ± SE, ^*^P < 0.05, ^**^P < 0.01, compared with control, ^##^*P <* 0.01, compared with CRO.

ERK1/2 phosphorylation, which plays a vital role in signal transduction associated with ENaC activity [34], was examined after treatment of H441 cells with 80 μM CRO. As shown in Figure 5A, CRO induced ERK1/2 phosphorylation by 30 min, and the impact of CRO on ERK1/2 phosphorylation was time-dependent (Figure 5B, *P* < 0.05, n = 3). The full-length blots/gels were presented in Supplementary Figure 3. Pretreatment with MAPK/extracellular regulated kinase kinase (MEK) inhibitor PD98059 (40 μM) for 30 min before exposure to CRO appeared to reverse the CRO-enhanced ERK1/2 phosphorylation, strongly suggesting that CRO eliminates ENaC activity by elevating the phosphorylation of ERK1/2 (Figure 5C and 5D).

### CRO increases oxidative stress in H441 cells

**Figure 6:**
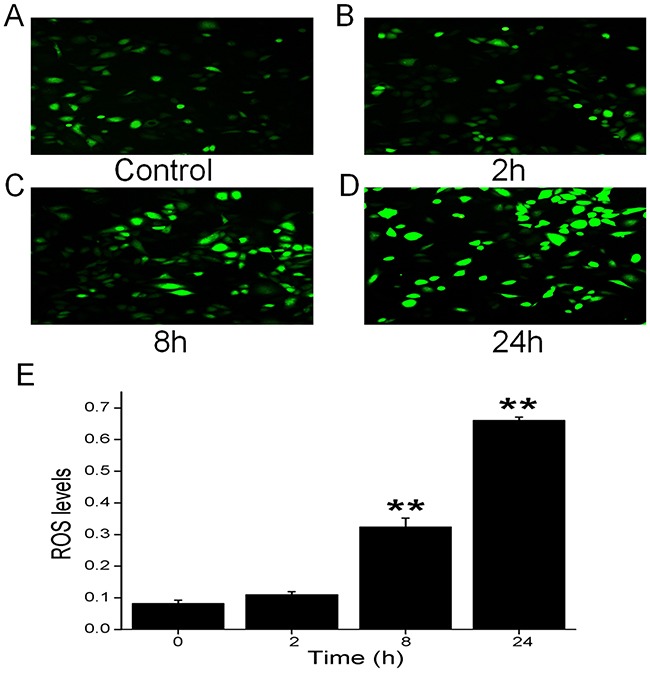
Effects of CRO on oxidative stress in H441 cells Production of ROS was measured by fluorescence microscopy using the fluorogenic substrate 2’,7’-dichlorofluorescein diacetate, which is oxidized to fluorescent 2’,7’-dichlorofluorescein. **(A)** Air-subjected control cell culture. **(B-D)** Cells exposed to 80 μM CRO for 2 h, 8 h, and 24 h. **(E)** The ROS levels were determined from the summary fluorescence intensity measured from the images of H441 cells (*n* = 25 in each group) that had been treated for different periods with CRO. Levels were corrected for background fluorescence. Data are shown as the mean ± SE, ^**^P < 0.01, compared with control.

Based on reports that cigarette smoke increases oxidative stress levels in the lung [35, 36], we hypothesized that CRO may have similar effects on the human distal lung cells. Therefore, we treated H441 cells with CRO at 0, 2, 8 and 24 h, and intracellular ROS levels were then detected by the dichlorofluorescein assay. Results confirmed ROS levels increased time-dependently in cells exposed to CRO (Figure 6A–6D), with significant differences detected in cells exposed for 8 h or 24 h compared to the unexposed cells (Figure 6E).

### CRO inhibits human αβγ-ENaC activity and interrupts membrane integrity in oocytes

**Figure 7:**
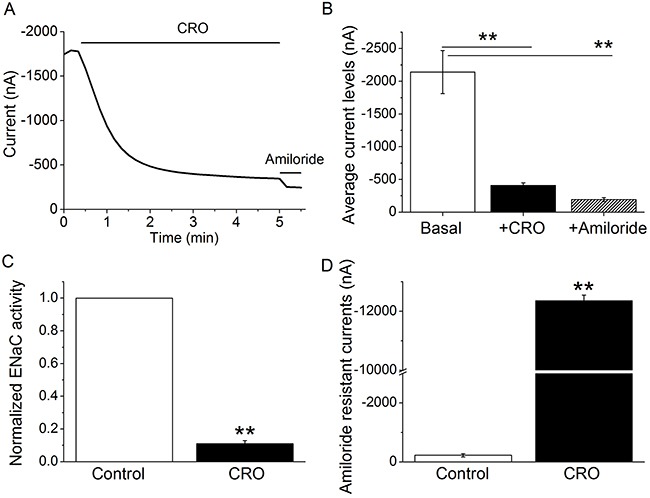
CRO alters the activity of heterologous human αβγ-ENaC channels expressed in oocytes and impairs properties of the plasma membrane Oocytes were perfused with CRO and amiloride continuously, and whole cell currents were monitored every 10 s. **(A)** Representative inward current traces in the presence of CRO and amiloride. Currents were digitized at -120 mV, and the period of drug application is indicated by solid horizontal lines. **(B)** Average currents at -120 mV (mean ± SE). ^**^P < 0.01 compared with basal level. **(C)** Normalized ENaC activity. Oocytes expressing human αβγ-ENaC were incubated with CRO for 24 h, and ENaC activity was evaluated. Amiloride-sensitive currents reflecting ENaC activity are defined as the difference between the total current and the amiloride-resistant current, with the basal amiloride-sensitive current set as 100%. **(D)** Average amiloride-resistant currents at -120 mV (mean ± SE), reflecting oocyte permeability. ^**^P < 0.01 compared with control. n = 8 from four different frogs.

To test the effects of CRO on human αβγ-ENaC, oocytes expressing the human subunits of ENaC were superfused with 80 μM CRO. Representative inward current traces in the presence of CRO and amiloride are shown in Figure 7A; the inward and whole cell currents were reduced by CRO and reached a stable plateau in a couple of minutes. In the presence of amiloride (10 μM), αβγ-ENaC activity was almost wholly inhibited (Figure 7B). Amiloride-sensitive currents, which reflect ENaC activity, are defined as the difference between the total current and the amiloride-resistant current, with the basal amiloride-sensitive current set as 100% control. The normalized ENaC activity at -120 mV following CRO administration was approximately 11% of the control group, a statistically significant change (Figure 7C, *P* < 0.01). These results suggest that CRO can rapidly inhibit human αβγ-ENaC activity. Next, oocytes expressing human αβγ-ENaC were incubated with CRO for 24 h. We analyzed the amiloride-resistant currents, which represent membrane permeability, and discovered a significant increase in the currents in CRO-pretreated oocytes compared to the control group (Figure 7D). These findings indicate that CRO leads to an augmentation in membrane permeability and interruption of membrane integrity in oocytes.

### ERK1/2 phosphorylation involved in CRO-reduced mouse alveolar fluid clearance *in vivo*

**Figure 8:**
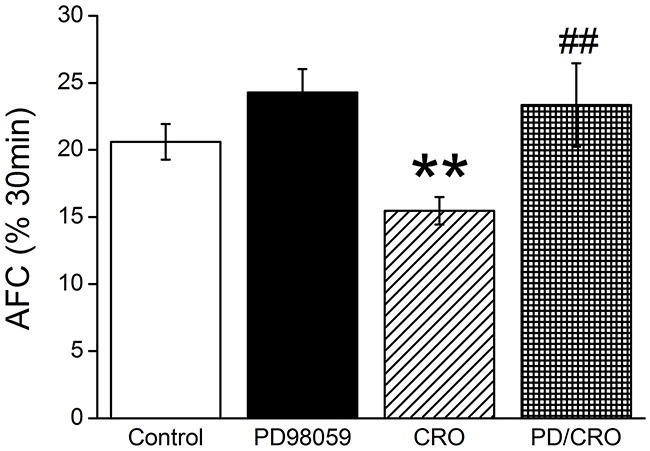
ERK1/2 phosphorylation involved in CRO-reduced mouse alveolar fluid clearance *in vivo* Anesthetized mice were intratracheally instilled with 5% bovine serum albumin dissolved in physiologic saline solution, administered alone for the control group (Control) or containing PD98059 (40 μM), crotonaldehyde (CRO, 80 μM), or both (PD/CRO) for exposed mice. The reabsorption rate was computed as the percentage of instilled volume after 30 min (% 30min). Average AFC values are presented as mean ± SE, one-way ANOVA. ^**^P < 0.01, compared with control, ^##^*P <* 0.01, compared with CRO, n = 6-8.

To corroborate the speculation that the rapid decrease of ENaC-associated AFC by CRO was related with the ERK1/2 phosphorylation, we repeated the above AFC experiment with PD98059 (MEK inhibitor) alone and the co-administration of PD98059 and CRO (PD/CRO). As shown in Figure 8, intratracheal instillation of PD/CRO recovered AFC rate to 23.4 ± 3.1% (n = 6), which showed much different with CRO alone (*P* < 0.01, n = 8). The above evidence provided that PD98059 could reverse the CRO-reduced AFC, indicating that the phosphorylation of ERK proteins were involved in the rapid decrease of AFC by CRO.

## DISCUSSION

AFC appears to play an essential part in lung fluid balance in both physiological and pathological conditions, as studies have showed that patients suffering from acute lung injury or the more severe acute respiratory distress syndrome have dysfunctional alveolar fluid clearance [37]. Much evidence has confirmed that Na^+^ reabsorption is a vital means of maintaining lung fluid balance and that ENaC plays a key role in Na^+^ reabsorption [1, 3, 38–41].

Our analyses revealed that that CRO can reduce AFC in mice. Additionally, since ENaC plays an indispensable role in AFC, we specifically examined the effects of CRO on ENaC function, using H441 cells. The H441 cell line was derived from human Clara cells found in the bronchiolar epithelium and has been used to study lung ENaC for the past two decades [32]; compared with primary cells, the cell line does not have the between-donor variances that can complicate the study of lung ENaC function following exposure to hormones, pollutants, and other substances. Furthermore, although H441 are cancerous cells, we believe they are an appropriate model for studying ENaC function as the airway epithelial cells of smokers can also be neoplastic. We found that CRO can inhibit the amiloride-sensitive Isc of H441 cell monolayers dose-dependently, strongly suggesting that CRO inhibits Na^+^ transport via suppressing lung ENaC. In our study, 80 μM CRO was chosen based on previous studies [36, 42] and from the IC_50_ value calculated in our Ussing chamber results, which amounts to one or two 2R4F Kentucky reference cigarettes [43]. We were excited to discover that both α- and γ-ENaC expression decreased at the protein and transcriptional level after CRO exposure.

ERK protein, consisting of ERK1 and ERK2, is a member of the MAPK family, which has a key part in signal transduction from surface receptors to the nucleus [44]. Previous research has shown that the ubiquitin ligase (Nedd4-2), which ultimately facilitates ubiquitination of ENaC by the increased ERK1/2 phosphorylation [34], controls ENaC channel activity at the apical membrane [45, 46]. Transforming growth factor-β1 has also been reported to reduce the expression of α-ENaC on apical membranes via the ERK1/2 pathway [47]. Additionally, the α,β-unsaturated aldehydes (acrolein and CRO) contained in cigarette smoke elicit interleukin-8 release in pulmonary cells through the MAPK pathway [48]. We explored the effect of CRO on protein phosphorylation in the ERK pathway and determined that the ERK1/2 phosphorylation level was elevated rapidly and strongly after exposure to CRO, whereas treatment with the MEK inhibitor PD98059 inhibited the CRO-induced phosphorylation of ERK1/2. The AFC data *in vivo* also showed that co-administration of PD98059 and CRO could reverse the CRO-reduced AFC, indicating that the phosphorylation of ERK proteins were involved in the rapid decrease of AFC in mice by CRO. Although we extracted total proteins for the phosphorylation and protein level analysis, the above findings implied that CRO may decrease ENaC activity sharply via elevating ERK1/2 phosphorylation levels in H441 cells. Howerver, in contrast to the inhibition of ENaC α-subunit expression in lung epithelial cells [22], Down and colleagues reported that cigarette smoke extract acutely increased ENaC activity in type I and II cells and increases AFC [49]. These inconsistent results might be due to the different methods for harvesting cigarette smoke extract and different responses between H441 and primary AT cells. The CSE contents (>7700 components), in strictly contrast to CRO, most likely led to the discrepancy. Importantly, our results could be a reasonable explanation of smoke inhalation-induced lung edema, a common clinical disorder, and the rationale for using aldehyde to prepare lung edema animal models.

**Figure 9:**
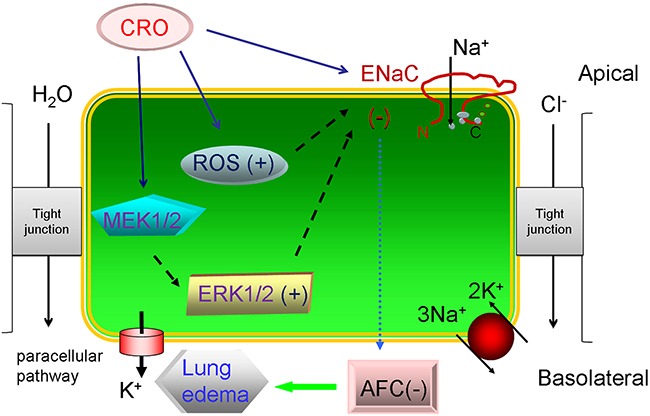
Schematic diagram summarizing the effects of CRO on alveolar fluid transport Cell exposure to CRO results in: reductions in ENaC activity via enhanced ERK1/2 phosphorylation, decreased transcription and translation of ENaC subunits, and elevation of levels of ROS products. These activities lead to the reduction of AFC and contribute to edematous acute lung injury.

ROS, in general, plays a key role in regulating alveolar ENaC and the maintenance of normal lung function [50]. It seems that there are different roles for ROS in the regulation of ENaC, both inhibitory and stimulatory. Elevated ROS could cause a decrease in ENaC activity, as seen in the PKC-α knockout lung [1, 51], as well as previous literature had reported that ROS had a stimulatory effect on ENaC activity [49, 52]. The discrete effects of extracellular and intracellular ROS on different cell and tissue models might regulate alveolar ENaC differently. A previous report has shown that ROS play a key role in CRO-induced apoptosis in alveolar macrophages [36]. Furthermore, extracts of cigarette smoke regulate lung ENaC and affect alveolar fluid balance via oxidant signaling [49]. Hence, we tested the effects of CRO on oxidative stress and determined that CRO can increase intracellular oxidation levels, as depicted in Figure 9. We speculated that the rapid decrease of Isc was associated with the specific inhibition of ENaC-associated function by CRO because only the amiloride-sensitive Isc, which mainly reflects ENaC activity in H441 monolayers, was affected. In contrast, the slow effect of CRO on oxidative stress reflects the overall trend of all channels in H441 cells, including non-ENaC components.

Xenopus laevis oocytes have minimal intrinsic channel activity and so have been widely applied to elucidate the electrophysiological features of channels through the injection of the corresponding cRNAs; ENaC subunit cRNAs are injected into the oocytes, and the biophysical properties and pharmacological profile of the heterologously expressed ENaC are similar to those of the native channels [53]. Another benefit of the oocyte expression system is that it eliminates potential complications from other native ion transport pathways, enabling us to observe the direct effects of CRO on human lung ENaC. The voltage clamp results using oocytes expressing human αβγ-ENaC indicated that CRO exposure markedly decreased ENaC activity and augmented the membrane permeability of oocytes.

## CONCLUSIONS

Taken together, our findings illustrate that CRO acutely reduces ENaC activity and that this reduction occurs at least partly via enhancing ERK1/2 phosphorylation; long-term exposure to CRO decreases the transcription and translation of ENaC subunits, and increases membrane permeability as well as the levels of cellular ROS products. These findings enhance our understanding of how CRO induces edematous acute lung injury.

## MATERIALS AND METHODS

### Animals

All experiments involving C57 mice and *Xenopus laevis* were performed according to the guidelines and regulations of Animal Care and Use Committee and all experimental protocols were approved by China Medical University and the University of Texas Health Science Center at Tyler, respectively. Animals were kept under pathogen-free conditions.

### Air-liquid cell cultures

The human distal lung epithelial cell line NCl-H441 was obtained from the American Type Culture Collection (ATCC) and cultured as previously described [54, 55], using RPMI-1640 medium (ATCC, Manassas, VA) supplemented with 10% fetal bovine serum (Gibco, Waltham, MA), 2 mM L-glutamine, 10 mM HEPES, 1 mM sodium pyruvate, 4.5 g/l glucose, 1.5 g/l sodium bicarbonate and antibiotics (100 IU/ml penicillin and 100 μg/ml streptomycin). Cells were incubated in a humidified atmosphere of 5% CO_2_ and 95% O_2_ at 37°C. For Ussing chamber assays, cells were grown in Costar Snapwell culture cups, until reaching confluency at 24 h, and then the culture medium was changed every other day for air-liquid interface cultures. We measured transepithelial resistance with an epithelial volto-hmmeter (WPI, Sarasota, FL) and selected the confluent filters for measuring Isc levels with resistance > 500 Ωcm^2^.

### Measurements of *in vivo* alveolar fluid clearance (AFC) in mice

AFC was examined *in vivo* as described previously [1, 56]. Briefly, 8- to 10-week-old C57 male mice, weighting 20 to 25 g, were anaesthetized with diazepam (17.5 mg kg^−1^, intraperitoneally), followed 6 min later by ketamine (450 mg kg^−1^, intraperitoneally), and were then placed on a heating pad. The trachea was exposed and cannulated with a trimmed 18-gauge intravenous catheter, which was then connected to a mouse respirator (HX-300, Chengdu Taimeng Technology Co. Ltd, Chengdu, China). Mice were ventilated with 100% O_2_ with a 200 μl tidal volume (8-10 ml kg^−1^) at 160 breaths min^−1^. A stock solution of 5% BSA, which was fatty acid-free and contained isosmotic NaCl, was prepared. The anesthetized mice were kept in a left-lateral position on a heating pad, and then 200 μl of the prepared BSA solution containing CRO (80 μM) and/or amiloride (1 mM) was instilled *via* the tracheal cannula, followed by 300 μl of room air to clear dead space. After instillation, mice were ventilated for a 30 min period, and then the alveolar fluid was aspirated. We measured the BSA content of the alveolar fluid using the coomassie brilliant blue method. AFC was calculated as follows: AFC = (Vi-Vf)/Vi×100, where Vi is the initial volume of instillate and Vf denotes the final alveolar fluid. Vf = (Vi×Pi)/Pf, where Pi and Pf represent protein concentration of the instilled solution and collected fluid, respectively.

### Ussing chamber assay

Measurements of transepithelial resistance and Isc were performed as previously described in H441 monolayers [32]. Briefly, H441 monolayers were mounted in Ussing chambers (Physiologic Instruments, San Diego, CA) and bathed on both sides with a solution (pH 7.3~7.4) including 120 mM NaCl, 25 mM NaHCO_3_, 3.3 mM KH_2_PO_4_, 0.83 mM K_2_HPO_4_, 1.2 mM CaCl_2_, 1.2 mM MgCl_2_, 10 mM HEPES and either 10 mM mannitol (apical compartment) or 10 mM glucose (basolateral compartment). Osmolality was between 290 and 300 mOsm/kg for either side of the bath solution. Both sides of the bath solutions were continuously bubbled with 95% O_2_ and 5% CO_2_ mixed gas at 37°C. We measured the Isc levels with 3 M KCl, connected on both sides to Ag-AgCl electrodes filled with 4% agarose salt bridges. H441 monolayers were short-circuited to 0 mV. A 10 mV pulse with a duration of 1 s was given every 10 s to measure transepithelial resistance. Data were collected using the Acquire and Analyse 2.3 software.

### Treatment of H441 with CRO

For analyzing the expression of ENaC at the protein and transcriptional level, we treated the cells at a concentration of 80 μM for various time periods ranging from 0 to 24 h. To analyze H441 protein phosphorylation, cells were allowed to reach approximately 90% confluency and were then starved in serum-free medium overnight before exposure to 80 μM CRO for 15, 30, 45, 60 and 90 min. PD98059 is a cell permeable, non-competitive, reversible inhibitor of MEK, a protein kinase upstream of p44/42 MAPK. In some experiments, H441 cells were pretreated with PD98059 (Beyotime, Shanghai) 40 μM for 30 min prior to CRO treatments.

To detect the intracellular ROS level, cells were incubated at 37°C with 80 μM CRO for 24 h, and ROS levels was analyzed according to the manufacturer's instructions for the Reactive Oxygen Species Assay Kit (Beyotime, China).

### Western blot analysis

H441 cells grown in 6-well plates were pretreated with CRO, and then washed twice with PBS and harvested for immunoblot assays. After separation on SDS-PAGE (10% polyacrylamide gels), proteins were transblotted onto PVDF membranes (Invitrogen, Waltham, MA). The membranes were blocked in 5% BSA in 1×TBS containing 1% Tween (TBST) at room temperature for 1 h and then incubated with primary antibody at a dilution of 1:2,000 in 5% BSA at 4°C overnight. Primary antibodies were used to detect ENaC subunits α-ENaC (PA1-920A; Thermo Fisher, Waltham, MA) and γ-ENaC (ab3468, abcam), ERK1/2 (4695; Cell Signaling, Danvers, MA), phosphoERK1/2 (4370, Cell Signaling), and β-actin (sc-47778; Santa Cruz Biotechnology, Santa Cruz, CA). The membranes were washed three times with 1×TBST for 10 min intervals before being incubated with horseradish peroxidase-conjugated goat-anti-rabbit or goat-anti-mouse secondary antibody at a dilution of 1:2,000 in 5% BSA. The image was developed by ECL kit after washing membranes three times for 10 min intervals, and data were collected using the Image J program.

### Real-time polymerase chain reaction (PCR)

For real-time PCR, H441 cells were washed with PBS twice after exposure to CRO for different time periods, and total RNA was then extracted by TRIzol reagent (Invitrogen, Waltham, MA) according to the manufacturer's instructions. RNA concentration was calculated by spectrophotometry at 260 nm. The specific oligonucleotide primers for the real-time PCR analysis of the α- and γ-ENaC subunits were as following: α-ENaC, 5’-AAC AAA TCG GAC TGC TTC TAC-3’ (sense), 5’-AGC CAC CAT CAT CCA TAA A-3’ (antisense); γ-ENaC, 5’-GCA CCG TTC GCC ACC TTC TA-3’ (sense), 5’-AGG TCA CCA GCA GCT CCT CA-3’ (antisense); GAPDH, 5’-AGA AGG CTG GGG CTC ATT TG-3’ (sense), 5’-AGG GGC CAT CCA CAG TCT TC-3’ (antisense). Total RNA (1μg) was applied as a template for each reaction and reverse transcription was performed by a single cycle of 37°C for 15 min and 85°C for 5 s. This was followed by a single cycle of 95°C for 15 min, and then 40 cycles of 95°C for 10 s, 60°C for 90 s, 72°C for 90 s. Relative expression of ENaC mRNA was calculated using the 2^-Δ(ΔCT)^ comparative method, with normalization of each sample against expression of the endogenous reference gene GAPDH.

### Measurements of ROS generation

Intracellular levels of ROS were quantified by the ROS sensitive fluorogenic substrate 2’,7’-dichlorofluorescein diacetate, which is oxidized to fluorescent 2’,7’-dichlorofluorescein intracellularly; the manufacturer's recommendation were followed (Beyotime, S0033). Briefly, cells were grown on 24-well plates to 70-80% confluence in RPMI-1640 medium, as described above, after which they were treated with CRO for up to 24 h. The cells were then washed twice with PBS and loaded with serum-free medium containing 10 μM 2’,7’-dichlorofluorescein diacetate for 20 min of incubation in 37°C in the dark. The cells were washed with serum-free medium three times to remove extracellular 2’,7’-dichlorofluorescein diacetate, and then analyzed by a Leica DMI 3000B inverted fluorescence microscope.

### Expression of human ENaC in oocytes

Oocytes were removed surgically from appropriately anesthetized adult female *Xenopus laevis* (Xenopus Express, Brooksville, FL), and cRNAs (α, β, and γ-human ENaC) were prepared as described previously [8]. In brief, frogs were placed under anesthesia by applying ethyl 3-aminobenzoate methanesulfonate salt (Sigma, Louis, MO) through a small incision in the lower abdomen, and then ovarian tissue was removed. Follicle cells were then removed and digested in Oocyte Ringer solution 2 calcium-free medium (82.5 mM NaCl, 2.5 mM KCl, 1.0 mM MgCl_2_, 1.0 mM Na_2_HPO_4_, and 10.0 mM HEPES, pH 7.5) with the addition of 2 mg/ml collagenase (Roche, Indianapolis, IN). Defolliculated oocytes were injected cytosolically with ENaC cRNAs (25 ng per oocyte) in 50 nl of RNase-free water, with a subunit ratio of 1α:1β:1γ, and incubated in half-strength L-15 medium at 18°C. Control cells were not treated with cRNA.

### Two-electrode voltage clamp assay

The two-electrode voltage clamp technique was used to record whole-cell currents 48 h after injection [55]. Oocytes were impaled with two electrodes filled with 3 M KCl, and the resistances ranged from 0.5-2.0 MΩ; the TEV-200 voltage clamp amplifier (Dagan, Minneapolis, MN) was applied to clamp the oocytes and record currents. Two reference electrodes were connected to the bath, which contained a bathing solution of ND96 medium (96.0 mM NaCl, 1.0 mM MgCl_2_, 1.8 mM CaCl_2_, 2.5 mM KCl, and 5.0 mM HEPES, pH 7.5) for continuous perfusion. Experiments were controlled by pCLAMP 10.1 software (Molecular Devices, Sunnyvale, CA), and a series of currents of -40, -100, and +80 mV was continuously monitored at intervals of 10 seconds. The current traces were acquired by stepping the holding potential to -120 mV while the monitoring currents were stable. Data were sampled at the rate of 200 Hz and filtered at 500 Hz.

### Statistical analysis

All results were presented as mean ± SE. One-way ANOVA computations were used to analyze the difference of the means for normally distributed data. The Mann-Whitney test was applied for nonparametric data. Significance levels were ^*^P < 0.05 and ^**^P < 0.01.

## SUPPLEMENTARY MATERIALS FIGURES AND TABLES



## Abbreviations

ENaC: epithelial sodium channels
MAPK: mitogen-activated protein kinase
CRO: crotonaldehyde
ERK1/2: extracellular signal-regulated protein kinases 1 and 2
ROS: reactive oxygen species
AFC: alveolar fluid clearance
BSA: bovine serum albumin
Isc: short-circuit currents
PCR: polymerase chain reaction
