# Supplementary material for: Novel mechanisms for crotonaldehyde-induced lung edema
Source: Oncotarget. 2017 May 12;8(48):83509–22. doi: 10.18632/oncotarget.17840 (PMC5663532; doi:10.18632/oncotarget.17840)
Supplement: Supplementary file 1 [file oncotarget-08-83509-s001.pdf]

Novel mechanisms for crotonaldehyde-induced lung edema

SUPPLEMENTARY FIGURES

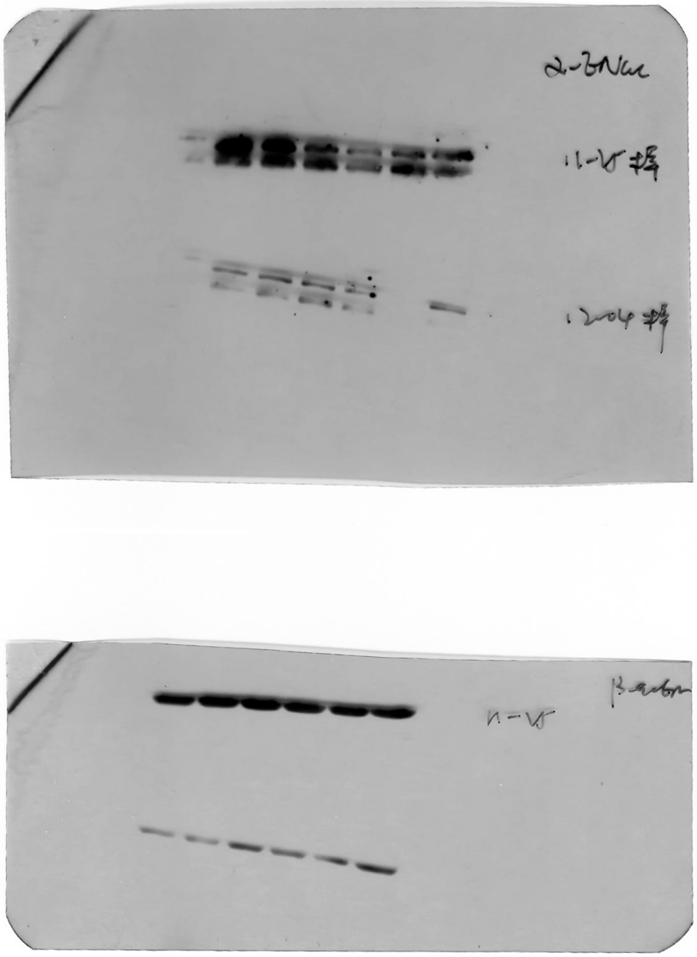

Supplementary Figure 1: The full-length blots/gels of α-ENaC protein extracted from H441 cells exposed to 80 µM crotonaldehyde for 0-24 h. Blots were immunostained with antibody to α-ENaC, or to β-actin as a loading control.

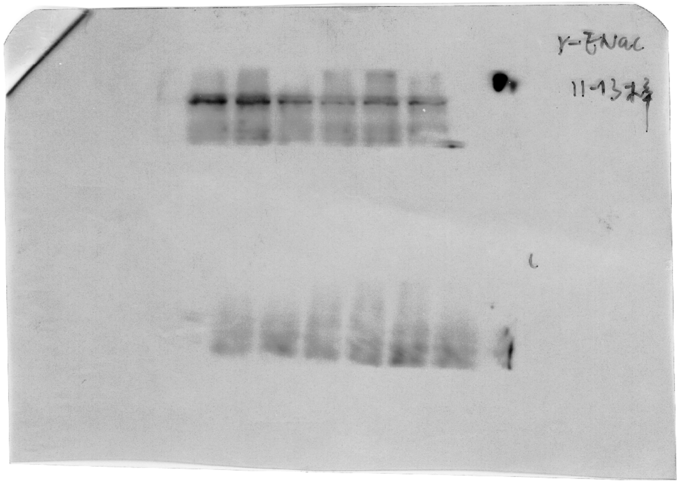

Supplementary Figure 2: The full-length blots/gels of γ-ENaC protein extracted from H441 cells exposed to 80 µM crotonaldehyde for 0-24 h. Blots were immunostained with antibody to γ-ENaC.

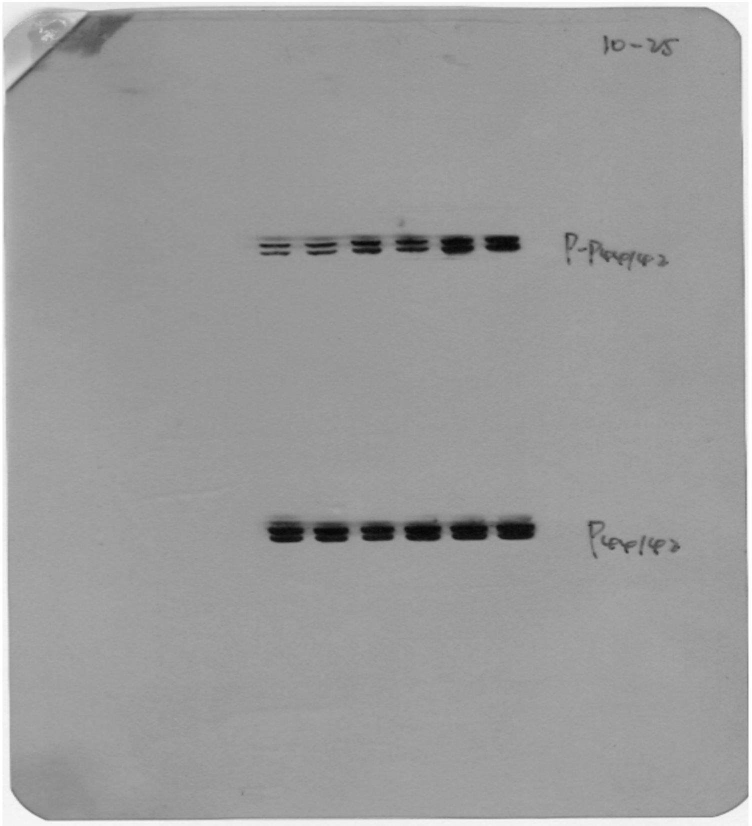

Supplementary Figure 3: The full-length blots/gels of phosphorylated ERK1/2 in protein extracted from H441 cells exposed to 80 µM crotonaldehyde for 0-90 min. Blots were immunostained with total ERK1/2 as a loading control.
